# Rare case of pelvic schwannoma mimicking intra-ligamenter uterine fibroid: A case report

**DOI:** 10.1016/j.ijscr.2022.107327

**Published:** 2022-06-20

**Authors:** Mila Maidarti, Yohanes Satrya Wibawa, Prini Diandara Garinasih, Tantri Hellyanti, Achmad Kemal Harzif, Kartiwa Hadi Nuryanto

**Affiliations:** a Division of Reproductive Endocrinology and Infertility, Department of Obstetrics and Gynecology, Faculty of Medicine, Universitas Indonesia, Jakarta 10430, Indonesia; b Yasmin IVF Clinic, Dr. Cipto Mangunkusumo General Hospital, Jakarta 10430, Indonesia; c Human Reproductive, Infertility and Family Planning Research Centre, Indonesia Medical Education and Research Institute (IMERI), Faculty of Medicine, Universitas Indonesia, Jakarta 10430, Indonesia; d Department of Anatomical Pathology, Faculty of Medicine, Universitas Indonesia, Dr Cipto Mangunkusumo General Hospital, Jakarta 10430, Indonesia; e Division of Oncology Gynecology, Department of Obstetrics and Gynecology, Faculty of Medicine, Universitas Indonesia, Dr Cipto Mangunkusumo General Hospital, Jakarta 10430, Indonesia

**Keywords:** CT, computed tomography, SCARE, Surgical CAse Report, CA-125, Cancer Antigen 125, MRI, Magnetic Resonance Imaging, AFP, Alpha-fetoprotein, SMA, Smooth Muscle Actin, CD117, cluster of differentiation 117, NF2, neurofibromatosis type 2, HE, hematoxylin and eosin, Pelvic schwannoma, Uterine fibroid, Case report

## Abstract

**Introduction and importance:**

Schwannomas are benign tumors originating from Schwann cells of nerve fibers. Pelvic schwannomas are extremely rare. Here, we present a case of a 48-year-old woman with a pelvic schwannoma imitating degenerating cystic leiomyoma.

**Case presentation:**

A 48-year-old woman presented with brown-yellowish bloody vaginal discharge, fever, abdominal enlargement, and pain. Abdominal ultrasound showed a homogeneous solid mass with a clear border separated from the uterus and left ovary. Computed tomography (CT) scan revealed a multilocular cystic mass with thick septa and solid enhancing component. Histopathological examination showed a mesenchymal tumor composed of cells with fine chromatin. The nuclei were oval or round, and some cells exhibited spindle and cigar-shaped nuclei. Tumor cells had an abundant amount of eosinophilic cytoplasm. Immunohistochemical examination demonstrated positive expression for S100 as specific staining for schwannomas. Mitosis was not found, and hyalinized blood vessels were observed.

**Clinical discussion:**

Compression by the tumor into the surrounding organs, such as the bladder and intestines, could cause difficulty with defecation and urination in patients. The absence of specific signs and symptoms can lead to a misdiagnosis of pelvic schwannoma. Surgery is the treatment of choice. It is difficult to establish a definitive diagnosis before surgery. Laparotomy approach was taken and a histopathological examination was completed to confirm the diagnosis.

**Conclusion:**

Unspecified pelvic pain with abdominal mass may suggest a rare tumor such as schwannoma. Transvaginal ultrasonography is incapable of reliably distinguishing between schwannoma and other tumors.

## Highlights

• Pelvic schwannomas are extremely rare.
• Pelvic schwannomas can be mistaken for uterine fibroids due to unspecific symptoms.
• It is challenging to establish a preoperative diagnosis of pelvic schwannomas.
• Surgery is the treatment of choice for pelvic schwannomas.

## 1 Introduction

Benign tumors originating from Schwann cells of nerve fibers are called schwannomas [1], [2], [3]. Predilection sites include the head, neck, mediastinum, and extremities [3]. Only about 1–3 % of schwannomas are located in the pelvis [4]. It is not easy to establish a preoperative diagnosis for schwannoma because there are no specific radiological characteristics and it is often misdiagnosed as a urological and gynecological related problem [5], [6]. Schwannomas tend to be asymptomatic, have a slow growth rate, and are difficult to be detected early. They are frequently discovered by chance. Necrosis, cystic degeneration, and calcification may also occur [1], [2], [3], [4], [7]. We report a case of pelvic schwannoma that was diagnosed as uterine myoma with cystic degeneration. This study has been reported in line with the Surgical CAse REport (SCARE) 2020 criteria [8].

## 2 Case presentation

A 48-year-old woman, P2A0, presented with brown-yellowish bloody vaginal discharge three months before admission. She complained of abdominal enlargement for the past 15 years and became much more prominent in the last five years, with fluctuating abdominal pain and fever for three months. There were also complaints of difficulty defecating and voiding. She has already had menopause for five years. There was a history of previous surgery due to uterine myoma, but the myoma could not be taken out due to severe adhesions. The patient had no prior drug or family history related to the disease.

Rectovaginal examination revealed a pelvic mass extended until two fingers below the umbilicus. The mass was with a smooth surface and limited mobilization. The cervix was hard to identify due to the expansion of the mass. Uterine sondage could not be performed due to the inability to expose the cervix. An increase in Cancer Antigen 125 (CA-125) level to 115.6 g/dL was found in the laboratory examination.

**Fig. 1:**
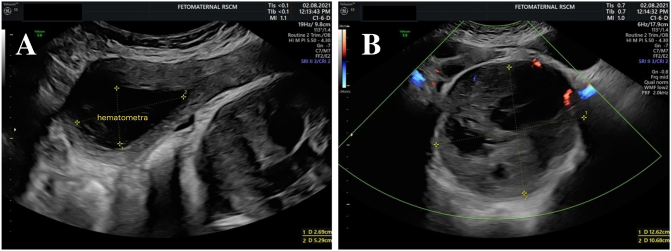
Abdominal ultrasound of the patient showing (A) hypoechoic mass sized 269 × 529 mm in the uterine cavity suspected for hematometra and (B) homogeneous solid mass sized 126 × 106 mm on the left adnexa suspected for myoma with cystic degeneration.

Abdominal ultrasound showed a 269 × 529 mm hypoechoic mass in the uterine cavity, suspected of infected hematometra. On the left adnexa, a homogeneous solid mass sized 126 × 106 mm with a clear border separated from the uterus and left ovary was found. The mass was suspected to originate from intraligamentery myoma with cystic degeneration, with a differential diagnosis of left ovarian fibroid (Fig. 1).

**Fig. 2:**
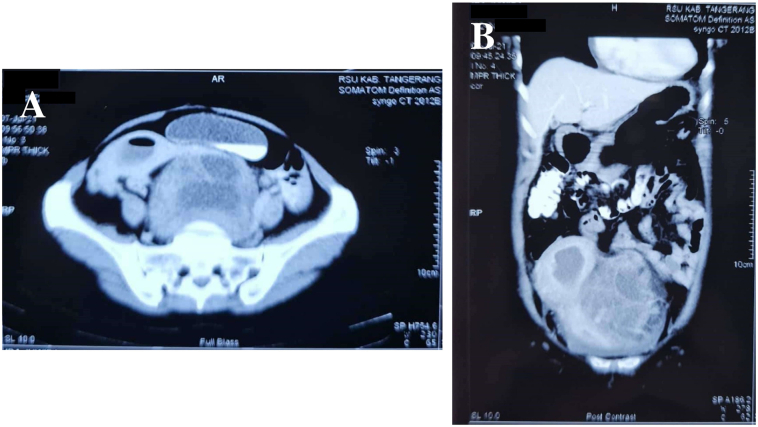
Abdominal CT scan of the patient in (A) axial and (B) coronal view showing multilocular cystic mass with thick septa and solid component intra lesion with enhancement pushing the uterus.

Abdominal and pelvic computed tomography (CT) showed a multilocular cystic mass with thick septa and solid component inside the lesion with enhancement, size 145 × 110 × 146 mm, clear border from uterus and bladder. Uterus pushed to the right superior lateral side, with the enlarged uterine cavity filled with fluid and air forming air-fluid level. There was no enlargement of para-iliac, para-obturator, and abdominal para-aortic lymph node (Fig. 2).

**Fig. 3:**
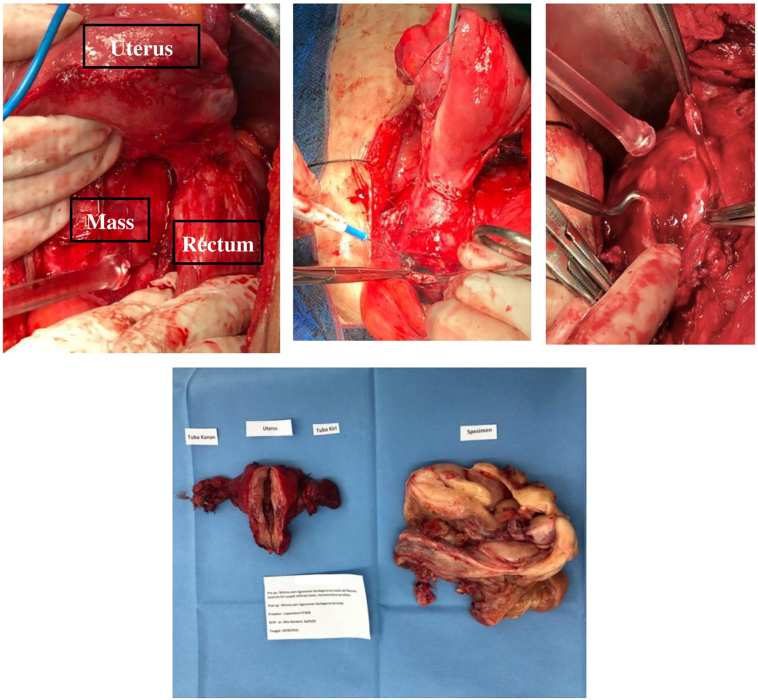
Gross morphology of the specimen taken out from the patient. A total hysterectomy with bilateral salpingo-oophorectomy was performed, followed by enucleation of the pelvic mass.

The patient underwent a laparotomy by a team of experienced obstetricians/gynecologist with a subspecialty in reproductive endocrinology and infertility and oncology gynecology. After the peritoneum was opened, intraligamentary mass was found adhered to the rectosigmoid compartment, with uterine corpus, both tubes, and ovaries within normal limits. Upon further exploration, a mass size of 150 × 130 × 120 mm was adhered to the bladder and pelvic side wall. We performed a total hysterectomy with bilateral salpingo-oophorectomy, followed by enucleation of the pelvic mass (Fig. 3).

**Fig. 4:**
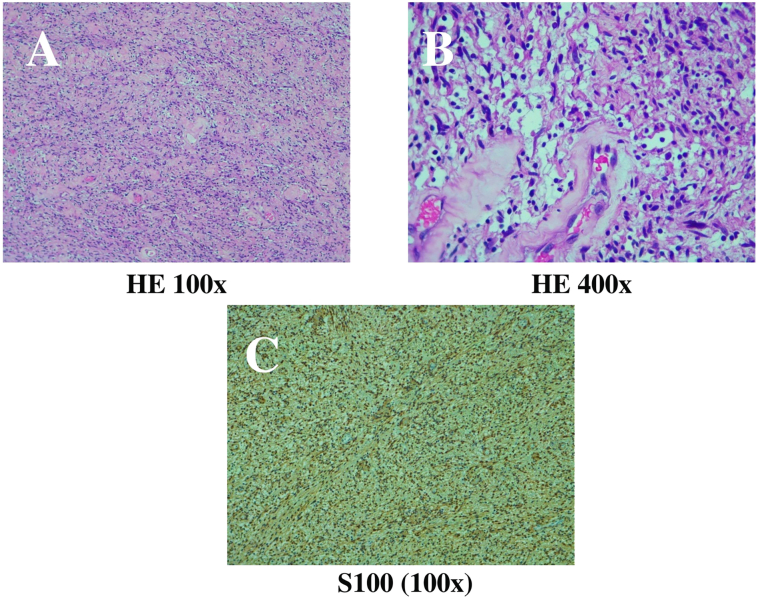
Histopathological examination using (A) Hematoxylin and eosin (HE) staining at 100× magnification and (B) 400× magnification revealed tumor cells with oval nuclei, spindle, some cigar-shaped, fine chromatin, eosinophilic cytoplasm. No mitosis was found. (C) Positive results with S100 with 100× magnification established a schwannoma.

Histopathological examination by an anatomical pathologist showed mesenchymal tumor tissue with hypocellular and hypercellular components. Tumor cells have oval, spindle, some cigar-shaped nuclei, fine chromatin, and eosinophilic cytoplasm. No mitosis was found, while hyalinized blood vessels were observed surrounded by tumor cells. The stroma was generally loose myxoid, and there was no lymph vascular invasion. The specimen was referred for immunohistochemical examination, which revealed a positive result for S100, a specific immunohistochemical staining for schwannoma (Fig. 4).

No significant complaints were reported in the postoperative period. Lower abdominal pain and vaginal discharge have been resolved. At the time of discharge, the pain had subsided significantly. Six months follow-up examination showed no recurrence and the patient is doing well.

## 3 Clinical discussion

Pelvic schwannomas are extremely difficult to diagnose preoperatively. They are often asymptomatic and found incidentally. Retroperitoneal schwannomas account for only 0.3–3.2 % of cases of benign schwannomas [9]. This tumor is more often found in men and people aged 20–50 years [1], [3], [7], [10], [11].

Our patient had been suffering from vaginal discharge, abdominal enlargement and pain. Difficulties in initiating defecation and micturition may be due to compression by the tumor into surrounding organs, such as the bladder and intestines. Slow urinary stream and radiating back pain can also happen. Symptoms may mimic endometriosis in the pelvis [4], [6], [7], [12]. Lower back and pelvic pain, urination and digestion problems may occur due to larger size and high likelihood of bleeding and spontaneous degeneration in retroperitoneal schwannomas, causing bladder and bowel compression [4], [6]. Compression from the tumor can result in sciatic nerve impingement [4]. The absence of specific symptoms may lead to a misdiagnosis of pelvic schwannomas [6].

On abdominal examination, there was a firm mass filling the lower abdomen. Rectal examination revealed a cystic mass two fingers below the umbilicus on the right adnexa. Schwannomas are usually encapsulated, growing independently, well-defined, firm, smooth-surfaced tumors with or without tenderness [1], [6], [13]. They tend to grow slowly and are non-aggressive neoplasms [14].

CT scan revealed a cystic multilocular left adnexa lesion with thick septa and solid intra-lesion component suspected of tubo-ovarian abscess. Lesions suggestive of infiltrating the sigmoid colon. The non-invasive criterion standard for diagnosing schwannoma has not yet been established [15]. No specific imaging features have been set for schwannomas, making the preoperative diagnosis difficult [1]. The size and location of the tumor can be estimated using ultrasonography, CT, and Magnetic Resonance Imaging (MRI) [3], [7], [10].

A well-defined, inhomogeneous cystic mass with well-demarcated cystic areas and a smooth outline are common CT features of schwannoma [2], [10]. Retroperitoneal schwannoma undergoes cystic changes more frequently [14]. The presence of cystic degeneration due to vascular thrombosis and adjacent hypocellular areas with more cellular sites give rise to an inhomogeneous schwannoma appearance on CT [10]. Compared to CT, MRI provides a better tissue differentiation [2], [10]. Low to moderate intensity on T1 and high intensity on T2 are the hallmarks of MR features of retroperitoneal schwannomas [2], [10]. Rim enhancement and target-like patterns and central enhancement can also be found on MR images [16]. However, these findings are not present in all cases and are not limited to schwannomas [13].

A preoperative biopsy is not required in operable tumors. Complete resection is the best method because of the cyst tumor risk of malignancy and infection [15]. Degenerative cells can be mistaken for malignancy due to cellular pleomorphism in degeneration sites [13]. Laboratory examination showed elevated CA-125 level, and the possibility of a malignant pelvic tumor had not been excluded. Pelvic schwannoma has no specific abnormal laboratory findings [1]. Teratoma and schwannoma can be differentiated by tumor markers Alpha-fetoprotein (AFP) and CA-125, whereas schwannoma is suspected if these tumor markers are negative [11].

Surgery is the treatment of choice. During surgery, there is a risk of damage to the bladder and ureters. Severe bleeding may occur with complete resection of a presacral schwannoma due to its proximity to the presacral venous plexus [7]. The position of the major blood vessels adjacent to the tumor may cause bleeding rather than the vascularity of the tumor itself [9].

Intraoperatively, we found intraligamentary mass adhered to the rectum, bladder, and pelvic side wall. Histopathological examination was consistent with schwannoma. No lymph vascular invasion was noticed. The Antoni A areas consist of spindle-shaped cells in the fascicles with palisade patterns (Verocay bodies), while the Antoni type B areas contain loose myxoid with an infiltrate of macrophages and are hypocellular [1], [2], [7], [17]. Other findings include cyst formation, hemorrhage, calcification, and hyalinization [1], [2]. There was no mitosis in the specimen, while hyalinized blood vessels were seen surrounded by tumor cells. This finding is common in schwannomas. Degenerative atypia and perivascular hemosiderin deposition may also be seen [7], [17].

The specimen sent for immunohistochemical examination showed positive results for S100, which corresponds to schwannoma. Other immunohistochemical stains that can be used include vimentin and neuron-specific enolase, while Smooth Muscle Actin (SMA) and cluster of differentiation 117 (CD117) will produce negative results [6]. Schwannomatosis and neurofibromatosis type 2 (NF2) have multiple schwannomas [17].

Benign adnexal masses, endometriosis, and primary ovarian neoplasms are gynecological cases resulting in chronic pelvic mass. Colorectal masses, hematoma, and soft tissue tumors are non-gynecologic causes [1], [3], [7], [18]. Misdiagnosis can occur in cases of extra-ovarian cysts that resemble ovarian neoplasms. The difficulty of diagnosis will increase in postmenopausal women due to the change in size and imaging of the ovaries [2]. Spindle cell fascicles and nuclear palisade can also be seen in leiomyomas resembling schwannomas [7]. Histologic and immunohistochemical findings in this study support the diagnosis of schwannoma.

The prognosis for schwannomas is good without early recurrence after surgery [1], [3], [7]. Rapid tumor growth, progressive pain, and severe muscle weakness can be signs of malignancy [12]. A malignant form of schwannoma may have high cellular components with active mitosis and invade the surrounding stroma [19].

## 4 Conclusion

In conclusion, unspecified pelvic pain with abdominal mass may indicate rare tumors such as schwannoma. Transvaginal ultrasonography is widely used in gynecological practice. However, it is incapable of reliably distinguishing between schwannomas and other tumors.

## Provenance and peer review

Not commissioned, externally peer-reviewed.

## Sources of funding

This research did not receive any specific grant from funding agencies in the public, commercial, or not-for-profit sectors.

## Ethical approval

This study is exempt from ethical approval.

## Consent

Written informed consent was obtained from the patient for publication of this case report and accompanying images. A copy of the written consent is available for review by the Editor-in-Chief of this journal on request.

## Author contribution

**Mila Maidarti**: Conceptualization, methodology, formal analysis, investigation, writing – original draft, writing – review and editing. **Yohanes Satrya Wibawa**: Formal analysis, investigation, writing – original draft. **Prini Diandara Garinasih**: Formal analysis, investigation, writing – original draft. **Tantri Hellyanti**: Conceptualization and supervision. **Achmad Kemal Harzif**: Methodology. **Kartiwa Hadi Nuryanto**: Conceptualization and supervision.

## Research registration

N/a.

## Guarantor

The Guarantor was also the corresponding author.

## Declaration of competing interest

The authors declare that they have no competing interests.
